# Discoveries Interview: Prof. Nikolaos G. Frangogiannis on the cardiac injury and repair processes

**DOI:** 10.15190/d.2014.12

**Published:** 2014-06-30

**Keywords:** Nikolaos G. Frangogiannis, cardiac injury, infarct, Albert Einstein College of Medicine of Yeshiva University, New York, Edmond J. Safra/ Republic National Bank of New York Chair in Cardiovascular Medicine

**Figure 1:**
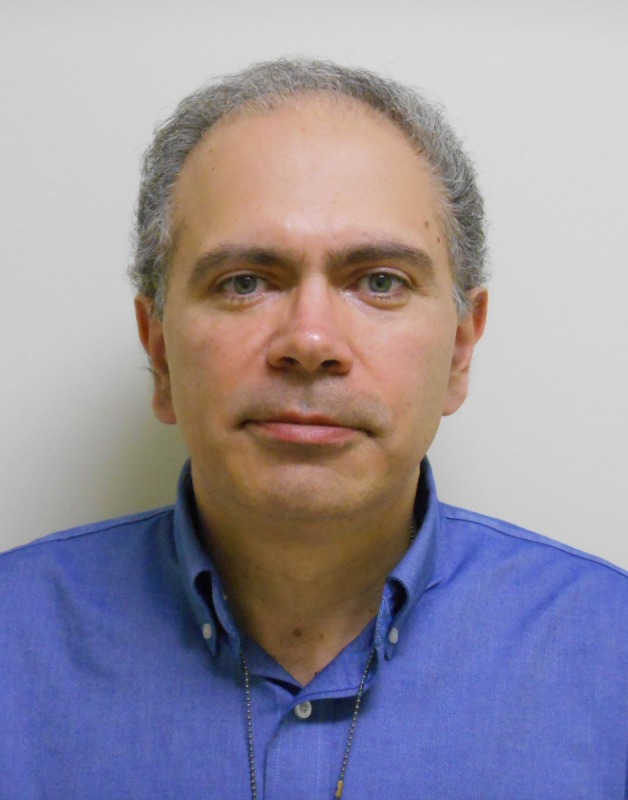
Professor Nikolaos G. Frangogiannis

**Prof. Nikolaos G. Frangogiannis** is a Professor of Cardiology, in the Department of Medicine at Albert Einstein College of Medicine of Yeshiva University, in New York, USA and the Edmond J. Safra/ Republic National Bank of New York Chair in Cardiovascular Medicine

*Dr. Frangogiannis* received his medical degree from the University of Athens Medical School, then completed residency training in Internal Medicine (Clinical Investigator Pathway), and a fellowship in Cardiology at Baylor College of Medicine (Houston TX). In 1999, he joined the faculty at Baylor, where he established his laboratory. In 2010, he relocated to Albert Einstein College of Medicine (Bronx NY), where he holds the Edmond Safra Chair in Cardiovascular Medicine.

## 1. Can you describe in simple words the cardiac injury and repair processes, when do they occur and why studying them is important?

The adult mammalian heart has negligible regenerative capacity and is very sensitive to injury; even relatively subtle changes in myocardial architecture can result in significant impairment of function. The most common form of cardiac injury in human populations is myocardial infarction, a condition due to sudden occlusion of a coronary artery, usually caused by rupture of an atherosclerotic plaque^1^. Cardiac myocytes are very sensitive to ischemia. 20-30 minutes after cessation of coronary flow, cardiomyocytes in the area subserved by the occluded artery become irreversibly damaged. Dying cardiomyocytes trigger a local inflammatory reaction that clears the infarct from dead cells and sets the stage for repair of the infarcted heart^2^. Unfortunately, humans (much like all other mammals) cannot regenerate their damaged heart. Thus, repair of the infarcted heart always results in formation of a collagen-based scar that protects the heart from a catastrophic event (such as rupture), but impairs the contractile capacity of the cardiac muscle. To make things worse, as the infarct heals, the heart “remodels”: the chamber enlarges and becomes more spherical, as cardiac dysfunction worsens^3^. Remodeling of the heart after infarction is associated with development of heart failure, arrhythmias and increased mortality. Myocardial infarction is a major cause of mortality and morbidity in both developed and developing countries; thus cardiac injury is responsible for many deaths and hospitalizations. Understanding the mechanisms of injury and repair is crucial in order to develop therapies for patients with heart disease.

Other forms of cardiac injury may result in remodeling of the heart and dysfunction, even when cardiomyocyte death is not prominent. For example, when the heart pumps against a high pressure (such as in hypertension, or in some forms of valvular disease), cardiac fibroblasts are activated, cardiomyocytes become enlarged and the heart thickens. This type of “remodeling” is associated with increased stiffness of the heart muscle that does not relax properly during diastole and causes heart failure despite the preservation of systolic function.

## 2. How our knowledge on cardiac injury and repair processes evolved over the time?

In the early 20^th^ century our knowledge on cardiac injury and repair was limited to descriptive information on the pathology of myocardial infarction. Over the last thirty years, progress in biological sciences and the increased interest in myocardial pathophysiology (fueled by the high socioeconomic burden of heart disease) resulted in better understanding of the molecular signals and cellular effectors implicated in cardiac repair. Clearly, development of strategies for genetic manipulation of mice provided us with great tools to study the role of specific genes and molecular pathways in cardiac injury, repair and remodeling. Animal model experiments have identified important mediators involved in cardiomyocyte death and have revealed key molecular signals implicated in cardiac repair and fibrosis. However, despite this progress, translation of this information into novel effective therapies for patients with heart disease has been rather disappointing.

## 3. Why is translation so challenging?

There are several reasons for the translational failures. First, our understanding of the pathophysiology of cardiac injury, repair and remodeling is far from complete. These processes involve a myriad of interacting molecules and many distinct cell types; our current understanding of these interactions is limited. In some cases, species differences may limit the relevance of some observations in human disease. Second, although animal models are very useful in dissecting pathophysiologic pathways, their value in predicting success of therapeutic strategies is limited. In particular for myocardial infarction, the complexity of the clinical context cannot be simulated by an animal model. In an optimally-designed animal investigation, our goal is to simplify the context in order to test a specific hypothesis. We use age- and gender-matched animals with identical genetic background, undergoing a well-defined experimental protocol to study the effects of a specific genetic manipulation. This strategy is very powerful for dissection of pathophysiologic processes; however, there is no similarly effective approach to predict effectiveness of a pharmacologic intervention. In the clinic, differences in patient characteristics (age, gender, genetic background), the presence of comorbid conditions, treatment with other medications, etc. affect the pathophysiologic response. This complexity cannot be simulated in an animal model; attempts to do so often weaken the value of the animal study. Ultimately, even for therapeutic concepts that seem to be safe for the patients and are supported by a strong pathophysiologic rationale, success can only be tested in clinical studies. This represents a major challenge and requires expensive and coordinated efforts.

## 4. Can you describe in simple words your research focus and the major contributions to the field?

My lab studies the endogenous healing response of the heart following myocardial infarction, the cell types involved in repair and remodeling, and the molecular pathways that regulate the reparative process. We also investigate how the heart becomes fibrotic in response to injurious stimuli that, unlike infarction, do not cause sudden death of a large number of cardiomyocytes, such as pressure overload, or metabolic disease. Cardiomyocyte necrosis results in release of danger signals that trigger a local inflammatory reaction: we have identified some of the key molecular signals responsible for recruitment and activation of leukocytes in the infarct^4,5^. We have also discovered pathways that suppress inflammation following infarction; these STOP signals appear to be critical for protection of the infarcted heart from adverse remodeling^2^. The heart also contains an abundant population of fibroblasts that can be activated by a variety of stimuli and deposit matrix in the cardiac interstitium, increasing stiffness of the heart. Our lab explores the pathways implicated in fibroblast activation and their interactions with the extracellular matrix^6^.

## 5. What will the field look like in 5-10 years?

The “holy grail” in the field is regeneration of the infarcted human heart^7^. Theoretically, this could be achieved by reactivating developmental cardiogenic pathways in injured adult hearts, by reprogramming reparative cell types (such as fibroblasts), or by introducing progenitors capable of cardiomyocyte regeneration. From a practical perspective, successful implementation of such strategies will likely require much more than a decade. Because of the complexity of the response to myocardial infarction, progress in understanding the pathophysiologic mechanisms is relatively slow. Despite the optimism often reflected in the lay press, translation into therapy is even slower.

## 6. What advice do you have for young scientists?

*To challenge existing knowledge*. Especially, in the field of pathophysiology of disease, many established concepts and published observations are likely to be incorrect, considering the limitations of the supporting evidence. If you trust the validity and robustness of your data, you should not dismiss your observations even if they challenge established dogma, but rather make every possible effort to uncover the truth.

## References

[1] Libby Peter (2013). Mechanisms of acute coronary syndromes and their implications for therapy.. The New England journal of medicine.

[2] Frangogiannis Nikolaos G (2014). The inflammatory response in myocardial injury, repair, and remodelling.. Nature reviews. Cardiology.

[3] Pfeffer M. A., Braunwald E. (1990). Ventricular remodeling after myocardial infarction. Experimental observations and clinical implications. Circulation.

[4] Frangogiannis Nikolaos G. (2012). Regulation of the Inflammatory Response in Cardiac Repair. Circulation Research.

[5] Swirski F. K., Nahrendorf M. (2013). Leukocyte Behavior in Atherosclerosis, Myocardial Infarction, and Heart Failure. Science.

[6] Frangogiannis Nikolaos G (2012). Matricellular proteins in cardiac adaptation and disease.. Physiological reviews.

[7] Laflamme Michael A, Murry Charles E (2011). Heart regeneration.. Nature.

